# Multichannel ECG recording from waist using textile sensors

**DOI:** 10.1186/s12938-020-00788-x

**Published:** 2020-06-16

**Authors:** Milad Alizadeh Meghrazi, Yupeng Tian, Amin Mahnam, Presish Bhattachan, Ladan Eskandarian, Sara Taghizadeh Kakhki, Milos R. Popovic, Milad Lankarany

**Affiliations:** 1 Clinical and Computational Neuroscience, Krembil Research Institute, University Health Network, 60 Leonard Ave, Toronto, ON M5T 0S8 Canada; 2 Institute of Biomaterials & Biomedical Engineering (IBBME), University of Toronto, Toronto, ON Canada; 3 Myant Inc, Toronto, ON Canada; 4 Department of Systems Design Engineering, University of Waterloo, Waterloo, ON Canada; 5 Department of Materials Science& Engineering, University of Toronto, Toronto, ON Canada; 6 KITE Research Institute, Toronto Rehabilitation Institute-University Health Network (UHN), Toronto, ON Canada

**Keywords:** Wearable electronics, Sensor fusion, Textile sensors, Multichannel ECG, *R*-peak detection, Probabilistic algorithm

## Abstract

**Background:**

The development of wearable health monitoring systems is garnering tremendous interest in research, technology and commercial applications. Their ability of providing unique capabilities in continuous, real-time, and non-invasive tracking of the physiological markers of users can provide insights into the performance and health of individuals. Electrocardiogram (ECG) signals are of particular interest, as cardiovascular disease is the leading cause of death globally. Monitoring heart health and its conditions such as ventricular disturbances and arrhythmias can be achieved through evaluating various features of ECG such as *R*-peaks, QRS complex, *T*-wave, and *P*-wave. Despite recent advances in biosensors for wearable applications, most of the currently available solutions rely solely on a single system attached to the body, limiting the ability to obtain reliable and multi-location biosignals. However, in engineering systems, sensor fusion, which is *the optimal integration and processing of data from multiple sensors*, has been a common theme and should be considered for wearables. In recent years, due to an increase in the availability and variety of different types of sensors, the possibility of achieving sensor fusion in wearable systems has become more attainable. Sensor fusion in multi-sensing systems results in significant enhancements of information inferences compared to those from systems with a sole sensor. One step towards the development of sensor fusion for wearable health monitoring systems is the accessibility to multiple reliable electrophysiological signals, which can be recorded continuously.

**Results:**

In this paper, we develop a textile-based multichannel ECG band that has the ability to measure ECG from multiple locations on the waist. As a proof of concept, we demonstrate that ECG signals can be reliably obtained from different locations on the waist where the shape of the QRS complex is nearly comparable with recordings from the chest using traditional gel electrodes. In addition, we develop a probabilistic approach—based on *prediction* and *update* strategies—to detect *R*-peaks from noisy textile data in different statuses, including sitting, standing, and jogging. In this approach, an optimal search method is utilized to detect *R*-peaks based on the history of the intervals between previously detected *R*-peaks. We show that the performance of our probabilistic approach in *R*-peak detection is significantly better than that based on Pan–Tompkins and optimal-threshold methods.

**Conclusion:**

A textile-based multichannel ECG band was developed to track the heart rate changes from multiple locations on the waist. We demonstrated that (i) the ECG signal can be detected from different locations on the waist, and (ii) the accuracy of the detected *R*-peaks from textile sensors was improved by using our proposed probabilistic approach. Despite the limitations of the textile sensors that might compromise the quality of ECG signals, we anticipate that the textile-based multichannel ECG band can be considered as an effective wearable system to facilitate the development of sensor fusion methodology for pervasive and non-invasive health monitoring through continuous tracking of heart rate variability (HRV) from the waist. In addition, from the commercialization point of view, we anticipate that the developed band has the potential to be integrated into garments such as underwear, bras or pants so that individuals can use it on a daily basis.

## Background

The ultimate goal of the wearable technology is to enable continuous access to humans’ physiological states. This is achievable through real-time tracking of physiological signals that can capture bio-information underlying the users’ health status. Wearable health monitoring systems allow clinicians and caretakers to continuously monitor changes in the patient’s vital signs. For example, ECG monitoring can be used for tracking the health conditions of people suffering from ventricular disturbances, arrhythmias and other diseases like diabetes and Parkinson’s disease [1–3]. Wearable health monitoring, in turn, empowers patients to be active in the optimal management of their chronic or acute conditions [4, 5] and provides non-intrusive monitoring of at-risk groups [6]. Therefore, wearable systems for continuous health monitoring provide proactive, affordable, and personalized health care services to the general population, especially individuals in need [7, 8].

Despite the ever-increasing use and commercialization of wearable electronics, limitations are impeding the success and utility of existing products for health monitoring. Devices such as smart watches are limited to a single location on the body (e.g., the wrist) [9], thereby restricting the access to different types of biosignals which are detectable from multiple locations on the body. While some systems such as Holter monitor can detect signals from multiple locations on the body, they are often obtrusive to day-to-day activities due to the presence of wires and the need for a clinician to position gel electrodes on the body. In addition, the embedded sensors are uncomfortable and have limited longevity. Current wearables, such as the Polar Belt [10] (© Polar Electro) and Myo™ armband [11] (North™, Waterloo, Canada, formerly Thalmic Labs) are obstructive as they must be worn as an addition to an individual’s day-to-day attire. To address these challenges, multi-sensing and processing approaches like sensor fusion might offer a unique solution, specifically if they can be integrated into comfortable, wearable, and multi-purpose electrodes like textile sensors.

Smart or electronic textiles (e-textiles) are textile products capable of interacting with the environment and the users. The development of e-textiles is made possible through flexible textile circuitry, which paves the way for a truly unobtrusive and universal garment-based wearable devices. Textile sensors have been used to sense biopotential [12–14], temperature and humidity [15, 16], respiration [17], and pressure sensing [18–20]. As such, e-textiles present a unique opportunity for unobtrusive integration of different sensing modalities in multiple locations on the body. In recent years, numerous studies have looked into the effects of electrode position, size, and skin contact pressure (holding pressure) on signal quality [21–23]. In addition, other factors such as electrode to skin sensorial comfort, integration or construction techniques, and laundering/reusability need to be considered in the design, development and selection of textile electrodes for long-term ECG monitoring [24, 25]. Athos, Hexoskin, OMSignal and Hitoe are examples of textile-based electronic devices that can collect ECG signal from the torso. Silver-based conductive yarns, silicone-based electrodes, and conductive polymer coated fibers are used in these products to create the textiles electrodes [26–28].

In this paper, we describe the development and assessment of a textile-based multichannel ECG band that measures ECG from multiple locations on the waist. This band contains four knitted textile sensors. Two types of conductive yarns are selected, creating silver-based and carbon-based textile sensors. Using a data acquisition board designed in-house for multichannel textile sensing, we show that ECG signals can be reliably obtained from different locations on the waist where the shape of the QRS complex is reasonably similar to those recorded from the chest using traditional gel electrodes. As well, we develop a novel probabilistic approach for detecting *R*-peaks that enables heart rate variability (HRV) to be continuously monitored during different tasks, namely, *sitting*, *standing,* and *jogging*. Our investigation suggests that the developed textile-based band can be considered as the first step towards the development of sensor fusion methodology for pervasive and non-invasive health monitoring through continuous tracking of HRV from the waist. Although the developed waist band is used as a proof of principle in this paper, we anticipate that this textile-based band can be integrated into garments such as underwear, bras or pants.

The organization of this paper is as follows. In “Results”, we show that the *R*-peaks can be reliably detected from different locations on the waist using our textile-based ECG band. As well, the performance of the proposed algorithm for *R*-peak detection from textile sensors is compared with that of the conventional algorithms. Concluding remarks, discussions, and future directions are provided in “Discussion”. Finally, “Materials and methods” are provided in four sections, namely, textile sensors, multichannel ECG recording units, signal processing algorithm, and statistical tests.

## Results

In this paper, ECG signals were simultaneously recorded from four different locations on the waist using textile sensors (see "Materials and methods" for the exact locations of the sensors), as well as a reference ECG signal from the chest using gel electrode from 6 subjects. All the tests were conducted in accordance with a Research Ethics Board (REB) approved by the University of Toronto. All participants gave their consent to participate after being informed of the nature and objectives of the experiment. Data were recorded during two stationary statuses, namely, sitting and standing, each of which lasts for 2 min. Following the same protocol, ECG signals were recorded during jogging to compare the performance of the proposed algorithm for *R*-peak detection, i.e., History-Dependent Inverse Gaussian (HDIG), vs. conventional algorithms, namely, optimal-threshold and Pan–Tompkins (PT) [29]. The detected *R*-peaks were compared with those detected (simultaneously) from the chest using gel electrode (which is considered as the reference signal), and the accuracy (ACC) and F1 score were calculated (see Additional file 1: Appendix A for more details). The *ACC* and *F1*-*score* are the major statistics to quantify the quality of binary classifications (*R*-peak detection). Both measures lie within [0,1], where 0 and 1 represent the worst and best performances, respectively.

### Textile sensors are reliable for continuous detection of *R*-peaks from the waist

**Fig. 1:**
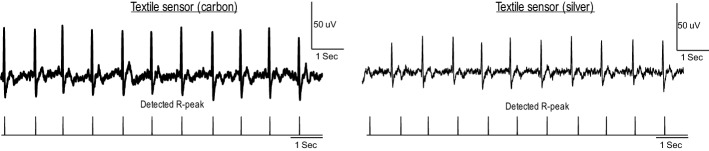
Two ECG signals recorded in the sitting status from the waist using carbon (left) and silver electrodes (right)

Figure 1 shows two examples of recorded ECG signals and detected *R*-peaks using silver and carbon electrodes. As can be seen in this figure, the recorded ECG signals from the waist using both silver and carbon textile sensors are reliable enough to accurately track the heart rate.

**Fig. 2:**
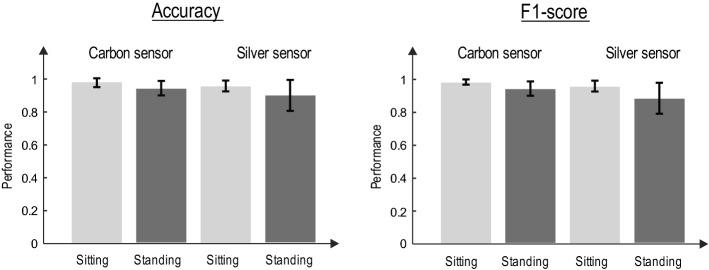
Performance of textile sensors in *R*-peak detection from the waist. The HDIG algorithm is used for *R*-peak detection. The accuracy (left) and F1-score (right) of the detected *R*-peaks in different states (sitting and standing) are calculated with respect to those detected by gel electrode (chest)

Figure 2 shows the performance of each textile sensor for *sitting* and *standing* statuses by the mean and standard deviation of ACC and F1-score of the detected *R*-peaks.

As it is obvious, the accuracy of detection of *R*-peaks from the waist using both silver and carbon sensors is comparable with that detected from the gel electrode (chest), confirming that the heart rate can be monitored from waist using textile electrodes in the stationary statuses.

### Proposed *R*-peak detection algorithm for textile-based recordings is robust to motion artifact

**Fig. 3:**
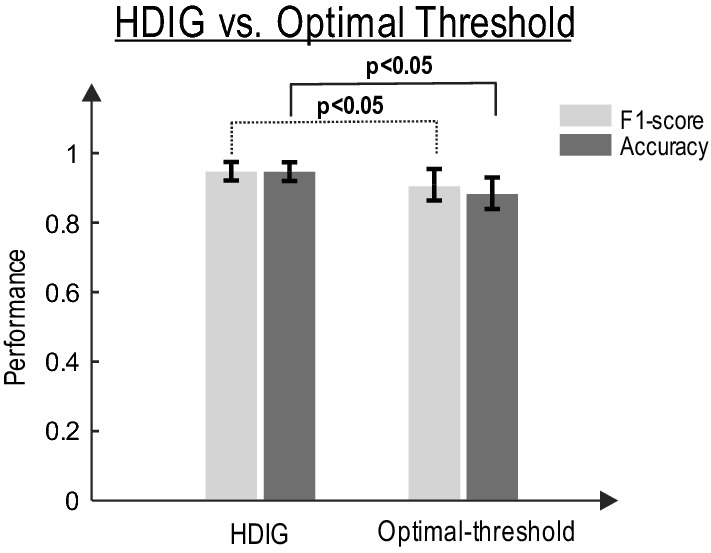
The detection performance of HDIG vs. optimal-threshold methods (see Additional file 1: Appendix B) for sitting and standing states. Both accuracy and F1-score measures of the HDIG method are significantly higher than that of the optimal-threshold method. One-way ANOVA test (F-distribution) is used, *p*-values for F1-score and ACC are, 0.014 and 0.004, respectively

Nearly all textile-based sensors induce slowly varying motion artifacts into the signal [30]. Although such artifacts can be reduced by the use of appropriate electronic circuits as well as wearable designs which maintain a consistent skin–electrode connection with enough pressure, the presence of motion artifact in textile sensors is inevitable (see [31–38] for other alternative ways to reduce motion artifact in textile sensors). This necessitates the use of effective signal processing algorithms. Here, we show that exploiting the HDIG algorithm for peak detection (i.e., the 4th step of the proposed algorithm, see "Materials and methods") significantly enhances the ACC and F1-score of heart rate. The performance of the HDIG method is compared to that of the optimal-threshold method for both sitting and standing statuses. Figure 3 shows that both accuracy and F1-score of the *R*-peaks detected by the HDIG method are significantly higher than those obtained by the optimal-threshold method (*p* < 0.05 for both measures).

**Fig. 4:**
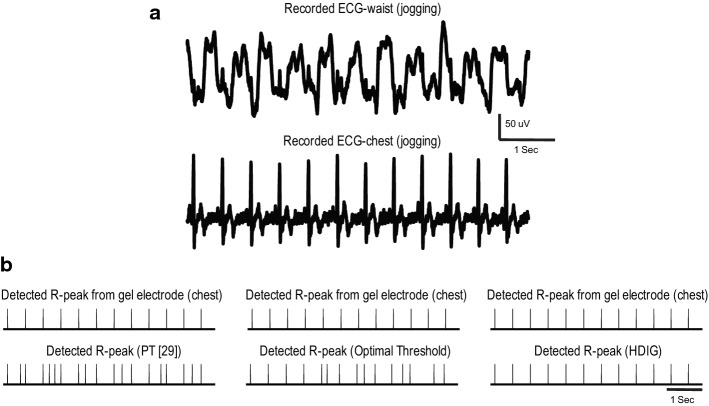
A segment of ECG signal during jogging, which is simultaneously recorded from the chest and the waist, is shown in **a**. *R*-peaks are detected in **B** using PT [29] (left), optimal-threshold (middle) and HDIG (right) methods. The *R*-peaks of the chest ECG is plotted as the reference

To further explore the robustness of the proposed probabilistic approach to motion artifact, the performance of this method is compared to that of the optimal-threshold and PT [29] methods during the *jogging* status in which motion artifact occurs more often. Figure 4a shows a segment of the recorded ECG from the waist during jogging. This signal is highly contaminated by motion artifact (slowly varying signal), and the *R*-peaks are barely detectable by visual inspection. The detected *R*-peaks using HDIG, optimal threshold, and PT [29] are shown in Fig. 4b. One can observe that all the *R*-peaks are correctly detected by the HDIG method, and neither false positive (FP) nor false negative (FN) is produced. However, the optimal-threshold and PT methods produce several FP and FN in this segment of the recorded signal.

**Fig. 5:**
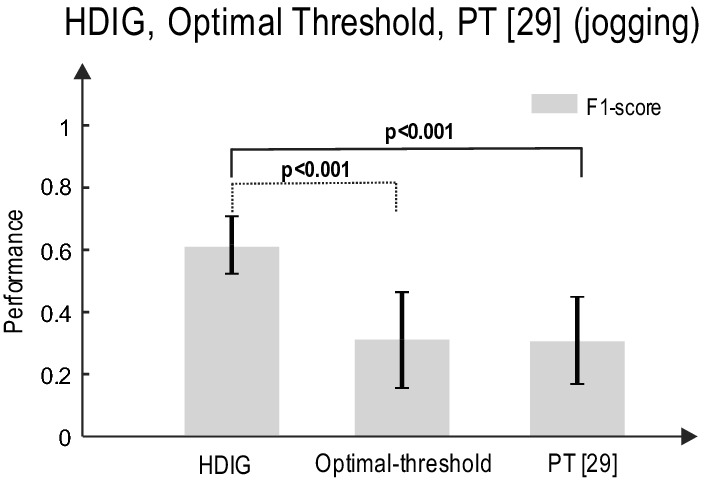
The *R*-peak detection performance of HDIG, optimal-threshold, and PT methods during jogging. One-way ANOVA test is used, *p-*values are 0.00062 and 0.00011 for HDIG vs. optimal threshold and HDIG vs. PT, respectively

In the *jogging* status where motion artifact is consistently larger than that in the stationary statuses (sitting and standing), the performance of HDIG is significantly more reliable than that of simple threshold (*p *< 0.001) and PT [29] (*p *< 0.001). Figure 5 shows the F1-score of the detected *R*-peaks using these algorithms during jogging (the results of both silver and carbon sensors are combined). It is to be noted that in the jogging status, due to the poor quality of the textile-ECG, true negative (TN) is high and therefore the true negative rate ($\frac{\text{TN}}{{{\text{TN}}\;{ + }\;{\text{FP}}}}$) is near 1. Therefore, we use F1-score to quantify the performance of the detected *R*-peak in the jogging state.

### Heart rate can be reliably monitored from different locations on the waist: implications for sensor fusion

**Fig. 6:**
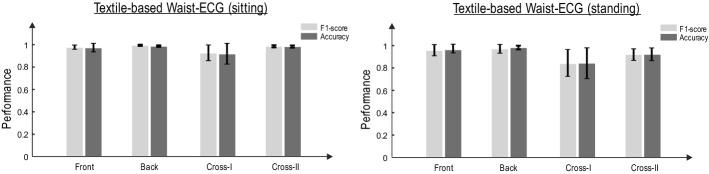
F1 score and accuracy of different locations, sitting and standing for the combined silver and carbon sensors (ANOVA test, for each state (sitting–standing–jogging), for 4 groups (1) back, (2) front, (3) cross-I and (4) cross-II

The ACC and F1-score of the detected *R*-peaks from various locations on the waist, namely, front, back, cross-I and cross-II (see "Materials and methods"), are evaluated in this section. The HDIG method is used to detect *R*-peaks from ECG signals recorded from each location on the waist. Figure 6 shows the ACC and F1-score of the detected *R*-peaks in the *sitting* and *standing* statuses for each location on the waist.

Although the accuracy of detected *R*-peaks is sufficiently high for all four locations, back and front sensors have relatively better performances. As shown in Fig. 5 (compared to Fig. 6), the performance of *R*-peak detection significantly reduces during the jogging status. However, this performance can be compensated by exploiting multiple sensors using sensor fusion. Although this is not the focus of this paper, the evidence of *R*-peak detection from multiple locations on the waist can be considered as the first step toward the development of sensor fusion methodologies to detect various features of ECG from the waist.

### Shape of QRS complex is preserved in the recorded ECG from the waist

**Fig. 7:**
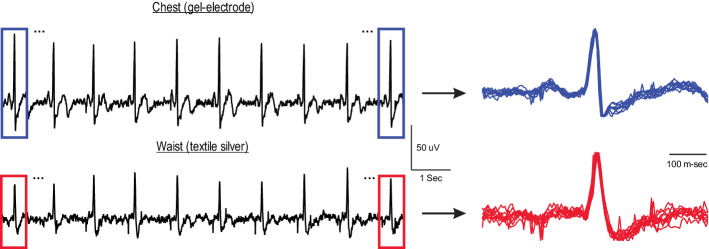
QRS segments obtained from ECG signals recorded from chest (top) and waist (bottom). The amplitude of each segment is normalized to its *R*-peak value

Despite the fact that *R*-peak detection is the major step in estimating the heart rate variability, other features in the ECG signals like the QRS complex are useful for classifying heart-related diseases. In this section, we quantify the similarity between the shape of the QRS complex recorded from the waist (using textile electrodes) and that from the chest (using gel electrode). The similarity measure can be written as:

1 $${\text{Similarity}} = 1 - \frac{{\frac{1}{\text{NL}}\mathop \sum \nolimits_{i = 1}^{N} \mathop \sum \nolimits_{m = 1}^{L} \left( {QRS_{i}^{\text{chest}} \left( m \right) - QRS_{i}^{\text{waist}} \left( m \right)} \right)^{2} }}{{\frac{1}{\text{NL}}\mathop \sum \nolimits_{i = 1}^{N} \mathop \sum \nolimits_{m = 1}^{L} QRS_{i}^{\text{chest}} \left( m \right)^{2} }},$$

where ${\text{QRS}}_{\text{i}}^{\text{chest}}$ and ${\text{QRS}}_{\text{i}}^{\text{waist}}$ indicate the *i*th QRS segment of the ECG signal recorded from chest and waist, respectively. All QRS segments are with the same length of L. As the similarity measure is normalized to the (average) energy of the QRS from chest (see [1]), this measure is positive and less than or equal to 1, where 1 represents the full match between ${\text{QRS}}_{\text{i}}^{\text{chest}}$ and ${\text{QRS}}_{\text{i}}^{\text{waist}}$. For each recorded ECG signal, the segments of the QRS complex are obtained by selecting 300 ms before and after the correctly detected *R*-peaks (i.e., true positive *R*-peaks). Figure 7 shows the selected QRS segments from a sample of recorded ECG.

**Fig. 8:**
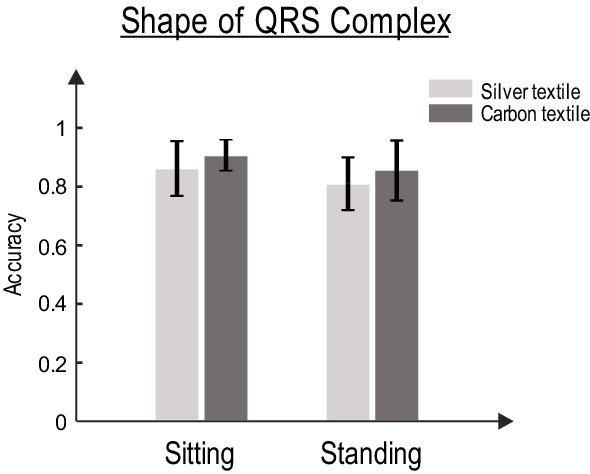
The QRS similarity measure for carbon and silver sensors

As can be observed from this figure, the shapes of the QRS complex of the chest and the waist are almost similar. The similarity measure is calculated for both types of textile sensors during stationary statuses. Figure 8 demonstrates that the QRS similarity measure (between waist and chest) is reasonably high (> 0.8) for both carbon and silver sensors. However, it is worth mentioning that the QRS duration appears to be longer in the waist-ECG compared to that in the gel electrode. Therefore, an accurate estimation of *P*-wave and other features of ECG such as PQ interval and T-wave using textile sensors might be compromised (see Discussion for further details on the limitations of textile sensors).

## Discussion

### Sensor fusion in wearable technology

Sensor fusion is the optimal integration and processing of data from multiple sensors that provide both redundancy and complementary data by maximizing information content. Sensor fusion reduces system’s sensitivity and uncertainty due to errors and artifacts, resulting in increased signal-to-noise ratios (SNR), enhanced system robustness and reliability, improved resolution and precision, and increased the dimensionality of measurements [6, 9, 39]. Sensor fusion has already received tremendous attention in automotive automation [40], mobile robot navigation [41], and target tracking [42]. In addition, sensor fusion techniques have been widely used in human movement analysis by using inertial measurement units (IMU) and respiration activity measurements given multiple physiological recordings. IMU-based sensor fusion have been used for pedestrian navigation (with GPS) [43] and human movement analysis in 3D orientations [44]. In respiration analysis, respiratory patterns have been analyzed with multiple ECG recordings [45] and data fusion techniques (e.g., modified Kalman filter [46]). Multi-sensing techniques, specifically in wearable sensors where the data can be highly contaminated by noise, facilitate the development of sensor fusion methods. Therefore, utilizing fusion techniques to interpret multi-sensor data from wearables is the next step towards optimizing health monitoring systems.

### Limitations of textile-based waist band for extracting various features of ECG

The quality of the waist-ECG signal and the accuracy of *R*-peaks depend on the body position. For example, in the *sitting* position, the quality of ECG was relatively similar for all different locations on the waist (no significant difference between different locations). This quality is reasonably high for all locations in *standing* position, however, the F1-score and ACC of “back” location were significantly higher than those of “cross-I” and “cross-II” locations (ANOVA *p*-value of F-statistic for both F1-score and ACC was < 0.05 for all pairwise comparisons). Despite the good enough similarity (> 0.8) of the QRS complex in the textile-ECG (waist) and gel-ECG (chest), the quality of various features of textile-ECG is compromised. For example, the QRS interval is prolonged and the amplitude of *P*-wave is reduced (not distinguishable from the baseline) in textile-ECG. Thus, the textile sensors might not be recommended when information of precise characteristics of ECG is to be inferred. In addition, textile sensors are highly sensitive (compared to the gel electrodes) to motions artifacts. In the jogging status, the quality of ECG and the performance of *R*-peak detection are influenced by large amount of motion artifacts.

Although the developed waist band provides a prototype for sensing ECG from multiple locations on the waist, further considerations on the textile sensors, electronics, and algorithms can improve the quality of ECG signals. Specifically, with respect to electronics, having textile-compatible electronics, e.g., placing pre-amplifiers subsequently after the textile sensors (i.e., active electrodes), can significantly improve the quality of ECG signals which in turn enhances the performance of *R*-peak detection algorithms. We investigate this line of research in our future studies.

### Implication of the multichannel ECG band for the development of sensor fusion methods

In non-stationary and time-varying body positions, i.e., the jogging status, a combination of locations might provide high-quality ECG signals. This capitalizes on the importance of optimal integration of multiple sensors, i.e., sensor fusion, for different body positions in wearable health monitoring systems. It is to be noted that all the ECG sensors in the multichannel ECG band provide a common signal. However, information underlying different features of the ECG signal, e.g., QRS complex, is not distributed uniformly between those sensors. Each sensor, in certain positions of the human body, might contain different information (of a common signal); thus, sensor fusion might be applied to better integrate data and infer information.

We anticipate that textile sensors and sensor fusion methodologies have the capability of non-invasively measuring and effectively processing a wide range of biometric signals. Therefore, e-textiles together with sensor fusion techniques can provide deep insights into an individual’s vital signs while increasing the quality, reliability, robustness, and precision of measurements. Systems utilizing this methodology can be used for widespread biological signal monitoring and feedback during the day-to-day activities and clinical settings [47–49].

## Conclusion

In this paper, we developed a textile-based multichannel ECG band that tracks heart rate changes from multiple locations on the waist. A data acquisition board was designed for multichannel recording through textile sensors. As well, we developed a novel probabilistic approach to detect *R*-peaks from noisy ECG signals recorded by the textile sensors. We showed that the *R*-peaks can be reliably detected from different locations on the waist, and the shape of the QRS complex is comparable with that recorded from the chest using traditional gel electrodes.

## Materials and methods

### Textile sensors (textile development)

**Fig. 9:**
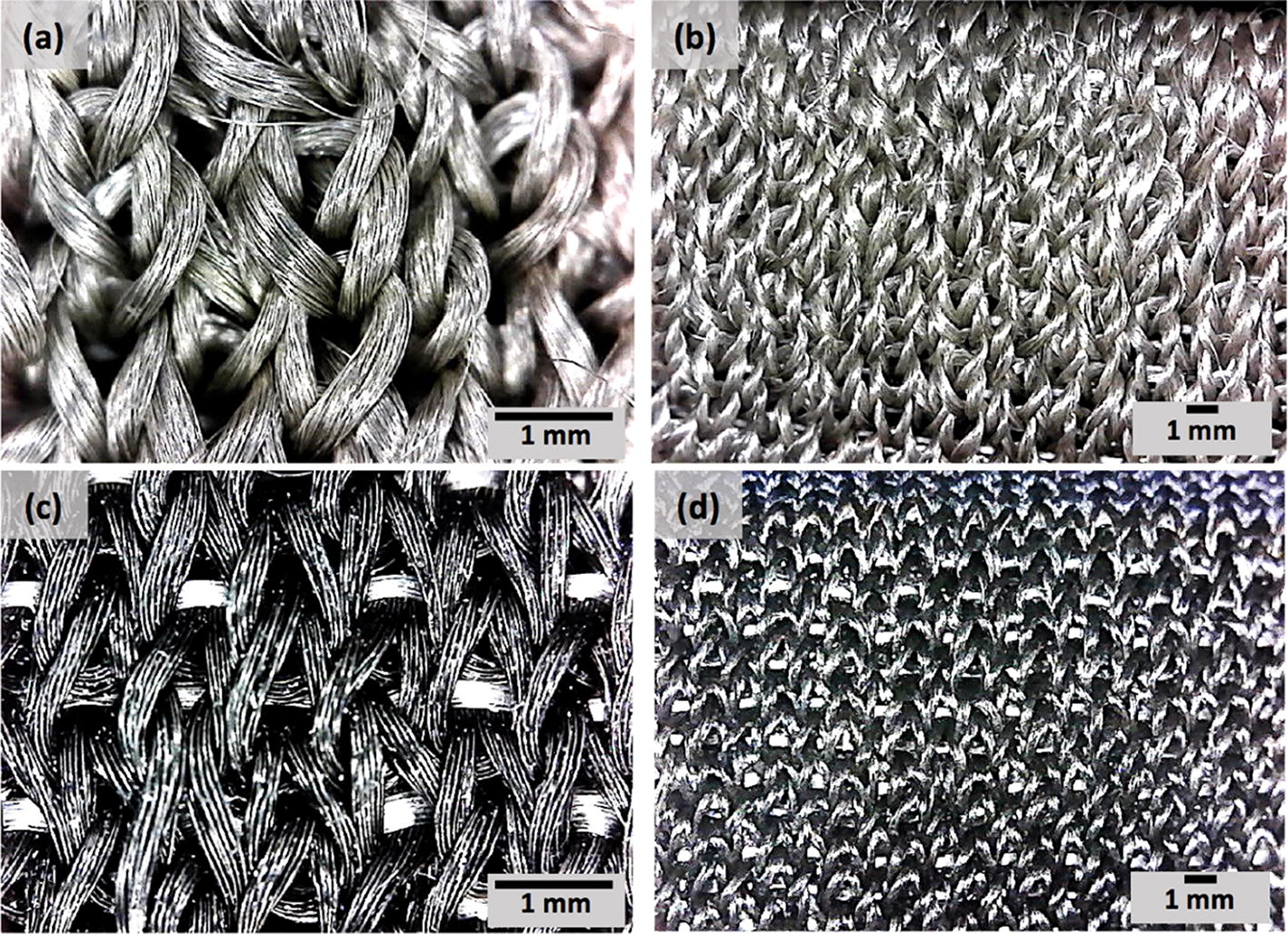
Photograph of textile sensors, silver (**a**, **b**), and carbon (**c**, **d**)

**Fig. 10:**
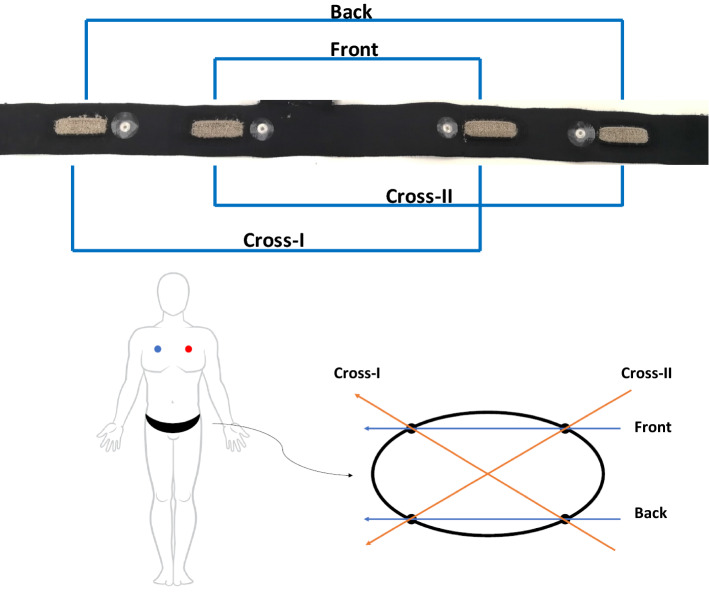
Photograph of the whole band. Schematic of gel electrode placement on chest and waist band electrode locations + vectors

Different types of materials have been used to produce conductive textile electrodes, these materials can be embedded into fabrics as conductive fibers, such as carbon, copper, or silver. In this paper, two different types of conductive textile electrodes were developed using silver-plated nylon yarns and carbon-coated nylon yarns. These yarns are the most frequently used conductive yarns in the smart textile industry. In order to compare the effect of these materials on ECG signal quality, textile-based multichannel ECG bands were knitted in a double jersey structure on a V-bed 18” gauge flat knitting machine. The textile electrodes were produced using Stoll flatbed knitting machines (Reutlingen, Germany) at Myant Inc. (Toronto, Canada). The electrode knitting structure can be seen in Fig. 9. Digital photographs were taken using Oitez USB microscope. Each band has 4 electrodes located at the iliac crest (×2) and across the frontal plane on the back (×2) (Fig. 10). The contact area of each textile electrode was 4.5 cm^2^ and the holding pressure of the textile electrodes in the band was 10 mmHg. The base band, which was the foundation for the electrodes, was made of a stretchable and washable fabric, regularly used in underwear/bra, waist/chest bands.

### Multichannel ECG recording unit

**Fig. 11:**
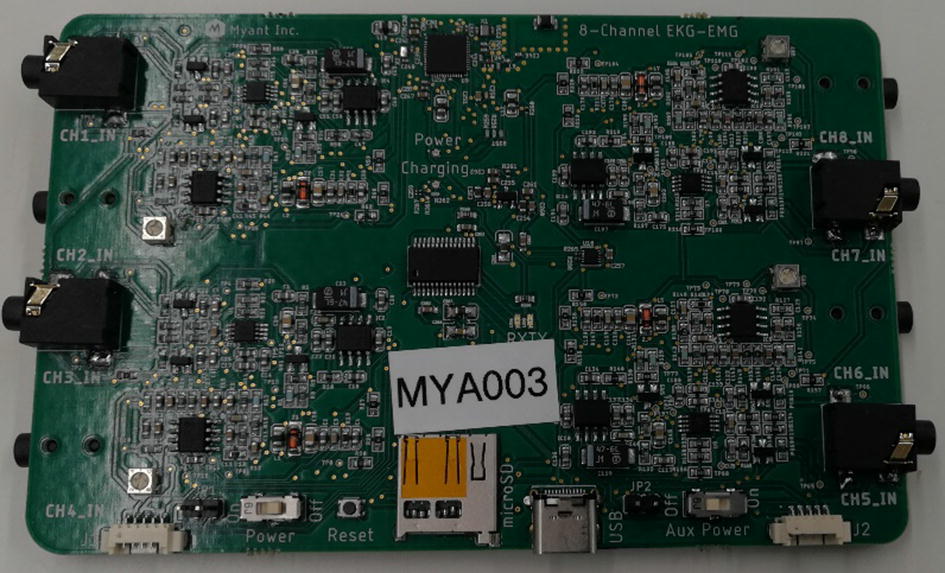
Photo of the designed board

Recording ECG with dry textile electrodes is generally challenging as the electrode–skin contact impedance is high, and it significantly varies between the electrodes [50]. This attenuates the signal before it can be amplified, and accordingly reduces the signal-to-noise ratio. In addition, the mismatch between electrodes results in high levels of interference noise on the signal [51]. In this paper, a custom-made biosignal recording system is developed to simultaneously record 8-channel ECG signals. Figure 11 shows an image of the developed 8-channel ECG measuring unit.

**Fig. 12:**
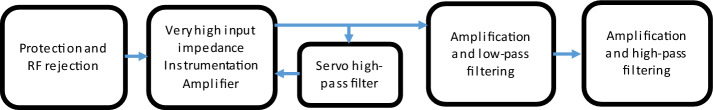
Block diagram 8-channel custom-made ECG recording circuit

The block diagram of the electronic circuit is depicted in Fig. 12.

As shown in Fig. 12, the first stage includes diodes for high-voltage protection, low-tolerance series resistors for limiting the current, and a differential passive low-pass filter for RF rejection (4 kHz cut-off frequency). The gain of the instrumentation amplifier was set to 10. An integrator circuit was implemented from instrumentation output to its reference as a negative feedback that acts as a high-pass filter with cut-off frequency of 0.16 Hz. The next amplifier has an adjustable gain between 5 and 100 and it works as a second-order low-pass filter with a cut-off frequency of 40 Hz. The last analog stage is a second-order high-pass filter (at 0.16 Hz) with a gain of 3.6. Therefore, the total high-pass filtering is of the third order. The cut-off frequencies were set based on the requirements for ECG monitoring which suggests maximum of 0.5 Hz for ECG monitoring applications and minimum 35 Hz for low-pass filtering [1]. The gain of all 8 channels was calibrated to be about 3000. This value was found appropriate to have a minimum quantization error while avoiding the amplifiers to saturate frequently due to motion artifact. The ground electrode was connected to the mid-rail driven by an operational amplifier. The outputs of all 8-channels go to the 12-bit resolution ADC of a STM32 microcontroller (multiplexing ADC) which transmits the data to a computer through Bluetooth. The circuit is powered with a 3.6 V battery and the sampling frequency was 200 Hz.

**Fig. 13:**
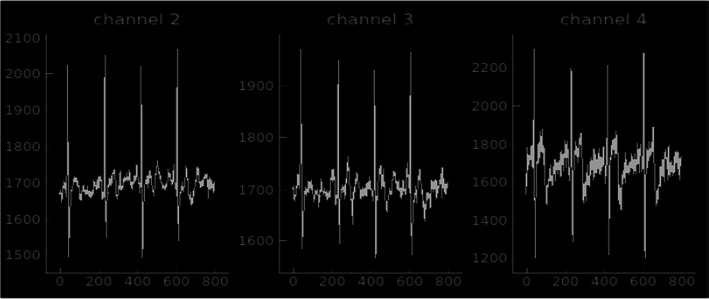
Screenshot of simultaneously recorded ECG signals (three channels in this example) using the developed recording unit. Note: channel 1 is grounded, and not shown in this figure

Three simultaneous recordings of ECG signals from chest (gel electrode, channel 4) and waist (silver textile electrode, channels 2 and 3) are plotted in Fig. 13.

### Signal processing (algorithm)

This part is a detailed explanation of the different steps used for the proposed algorithm.

## Background, prior works, and proposed algorithm

**Fig. 14:**
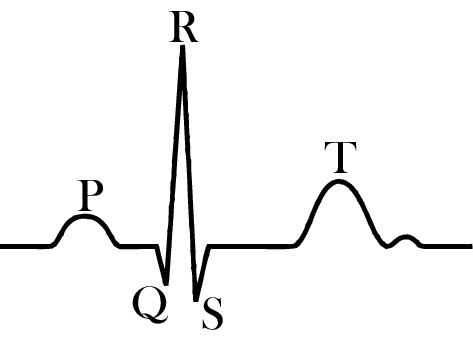
Most significant features of a typical ECG signal

**Fig. 15:**
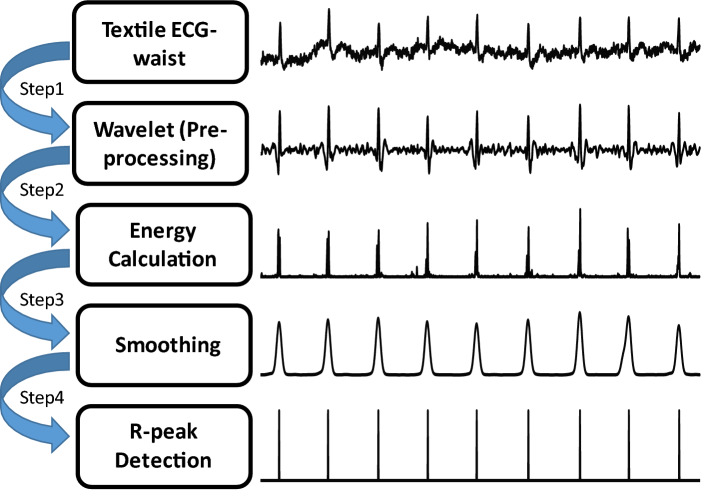
Schematic representation of the proposed algorithm

The ECG signal is nonlinear and non-stationary. ECG signal processing and feature extractions are more robust with nonlinear methods [52]. The most important features of an ECG signal (see Fig. 14) are: *P*-wave (atrial depolarization), T-wave (ventricular repolarization), and the QRS complex (ventricular depolarization). The QRS complex is the most prominent feature in an ECG signal, which has been widely used for the diagnosis of cardiac diseases and the assessment of the irregularities in the heart rhythm [53]. The accurate and efficient detection of *R*-peaks from the ECG signal is essential for further post-processing and classification of ECG signals. Due to the non-stationary nature of an ECG signal, and relatively high sensitivity of wearable sensors to motion artifacts and other external interferences, accurate detection of *R*-peaks from the waist is challenging in wearable technology. From a signal processing point of view [29], discrete wavelet transformation [54], empirical mode decomposition [55, 56], Hilbert transformation [57–59], and artificial neural network [60] are the most recognized methods for *R*-peak detection (see [53] for the details of each method). It is to be noted that almost all of these approaches characterize heart rate variability (equivalent to transient changes of R–R intervals, i.e., the interval between two adjacent *R*-peaks) as a deterministic continuous process—rather than a random (Poisson point) process where *R*–*R* intervals indicate the time difference between electrical impulses from the heart’s conduction system that represent ventricular contractions [3]. By incorporating Poisson point process model of the dynamics of *R*–*R* intervals, a novel probabilistic approach is developed in this paper to estimate the *R*–*R* intervals (and accordingly detect *R*-peaks) in real-time. Our proposed algorithm consists of four building blocks: (1) pre-processing (denoising), (2) energy calculations, (3) smoothing and (4) *R*-peak detection. The details of each block are described as follows. For better understanding the performance of each step, Fig. 15 shows the results of each step on a 5-s sample ECG signal.

### Pre-processing

We use a discrete wavelet transform for denoising and pre-processing the recorded ECG signals. The *sym6* wavelet is utilized to decompose the signal up to 5 levels. The fifth approximate coefficient is subtracted from the signal to eliminate the baseline drift (de-trending the signal). The *rigsure* threshold method is applied to the detailed coefficients to remove the high-frequency components. Finally, the de-noised signal is reconstructed from the thresholded detailed coefficients.

### Energy calculation

To enhance the representation of the *R*-peaks, the energy of the derivative of the pre-processed signal, *x*(*t*), is calculated:

2 $$E_{n} \left[ {x\left( t \right)} \right] = \left( {\frac{{d\left( {x\left( t \right)} \right)}}{dt}} \right)^{2},$$

where *En*[.] denotes the time-varying energy of the derivative of the pre-processed signal *x*(*t*).

The goal of this step is to amplify the effects of instances with abrupt changes (i.e., increase the signal-to-noise ratio $R = \frac{{{\text{local }}\;{\text{maximal }}\;{\text{peak}}}}{{{\text{nearby }}\;{\text{smaller}}\; {\text{peaks}}}}$), hence *R*-peaks will be detected with higher probability (with respect to the adjacent smaller peaks).

### Smoothing

To eliminate the fluctuations surrounding the local peaks in the calculated energy (see [2]), *En*[*x*] is filtered by a narrow Gaussian kernel (µ = 0, *σ*_kernel_ = 300 ms):

3 $$Z\left( t \right) = En\left[ {x\left( t \right)} \right]*N\left( {\mu , \sigma^{2} } \right),$$

where “*” indicates convolution, and *N*(.) is a zero-mean Gaussian kernel with a standard deviation of *σ*. This step helps better detection of *R*-peaks by smoothing the fluctuations near the peak of the energy signal.

### *R*-peak detection

An iterative probabilistic approach is developed—based on *prediction* and *update* strategies—to detect the peaks of *Z*(*t*). We use an optimal search method to detect peaks based on the History-Dependent Inverse Gaussian (HDIG) point process model of heartbeat intervals [3, 61]. Given any *R*-peak index *u*_*k*_, the RR interval is calculated based on HDIG model using the previously detected *R*-peaks within the 25-s interval preceding *u*_*k*_. The HDIG model provides precise probabilistic definitions of heart rate variability that can be updated at any desired time resolution. The time-varying parameters of the HDIG point process model are estimated by the local maximum likelihood estimation of instantaneous heart rate variability [3]. The search for the next *R*-peak, in *prediction step*, is performed within the interval $I = (u_{k} + {\text{RR(k)}} - a,u_{k} + {\text{RR(k)}} + a)$ for some chosen a (*a* = 300 ms in this work). The new *R*-peak, *u*_*k*+1_, is calculated, in the *update step*, as the maximum of *Z*(*t*) for *t ɛ I*. In fact, the HDIG model is incorporated to predict the interval within which the next *R*-peak occurs. Then, the detected *R*-peak in this interval updates the predicted *R*–*R* interval.

It is to be noted that we compare the performance of this probabilistic method (based on HDIG) in detecting *R*-peaks with that of the *optimal-threshold* method (see Additional file 1: Appendix B for details on this method). Therefore, steps 1–3 of our algorithm are the same for both methods.

### Statistical tests

The ANOVA tests are performed to compare different groups and to decide if they are significantly different. All ANOVA tests are performed for pairwise comparisons, i.e., two groups in each test. For example, for comparing the F1-scores of HDIG and that of PT, in the jogging state, all (jogging) samples from different electrodes across the waist are included. Then, the pairwise ANOVA test is performed: *F*-statistic is calculated and the corresponding *p*-values are shown.

For pairwise comparisons, the ANOVA test and *t*-test are almost equivalent. Both tests consider the difference between groups by comparing their mean and standard deviation. ANOVA test is more appropriate than *t*-test when ≥ 3 groups are to be compared. And, we choose ANOVA test over t-test to be consistent with the possible “comparisons for ≥ 3 groups” in the future work, e.g., it might be needed to compare multiple (≥ 10) electrode materials. Thus, ANOVA tests can be performed for all 10 groups, and if the results were significant, the group with the highest mean can be selected.

## Supplementary information

**Additional file 1.** Details of optimal-threshold method & statistical measures of R-peaks. This section includes two appendices, namely, A) statistical measures of the performance of detected R-peaks, and B) optimal-threshold method.

## Data Availability

The datasets recorded and analyzed during the present study are available from the corresponding author on reasonable request.

## Abbreviations

ECG: Electrocardiogram
PT: Pan–Tompkins
HDIG: History-dependent inverse Gaussian
