# Supplementary material for: Multichannel ECG recording from waist using textile sensors
Source: Biomed Eng Online. 2020 Jun 16;19:48. doi: 10.1186/s12938-020-00788-x (PMC7296680; doi:10.1186/s12938-020-00788-x)
Supplement: Supplementary file 1 — Additional file 1. Details of optimal-threshold method & statistical measures of R-peaks. This section includes two appendices, namely, A) statistical measures of the performance of detected R-peaks, and B) optimal-threshold method. [file 12938_2020_788_MOESM1_ESM.docx]

**Appendix**

1. *Statistical measures of the performance of detected R-peaks*

We consider the detected R-peaks in the ECG signals recorded by both textile and gel electrodes as the events. Apparently, a binary sequence of events and no-events can be made for each recorded ECG signal based on which the most common statistical measures, namely, true positive (TP), true negative (TN), false positive (FP) and false-negative (FN), are employed to calculate the performance of the proposed algorithm. The predicted peaks from the waist (textile electrode) and the chest (gel-electrode) are considered as “signal” and “ref”, respectively. To calculate the above-mentioned statistical measures, an event (equivalent to a detected R-peak) in “signal” is called TP if the underlying time index lies within [-25, 25]^msec^ of that associated with “ref”. This interval sets a precision-level for our classification (considering that the mean R-R interval for all subjects is about 750 msec, the classification error does not exceed $\frac{25ms}{RR interval}\approx\frac{25ms}{750ms}=3.3\%$). Accordingly, FP and FN denote the events that are incorrectly detected and missed (when compared to “ref”), respectively. The definition of TN for truly non-detected R-peaks is tricky. Since the sampling frequency is 200 Hz, the conventional definition of TN leads to a large number corresponding to all samples which are correctly detected as no-events. To eliminate that, the “no-event” corresponds to a set of all samples in “ref” which do not lie within the abovementioned interval. In better words, all the samples in “signal” that contains no R-peak and overlaps with “no-event” are counted as a TN. Figure S1. illustrates these statistical measures for a segment of “ref” and “signal”. In this figure, #TP = 6, #FP = 9, #TN = 4, #FN = 2, $sensitivity= \frac{TP}{TP+FN}=\frac{6}{8}=0.75$, $precision= \frac{TP}{TP+FP}=\frac{6}{15}=0.4$, $ACC= \frac{TP+TN}{TP+TN+FP+FN}=\frac{6+4}{6+9+4+2}=0.476$ and $F1=\frac{2TP}{2TP+FP+FN}=0.522$. *ACC* is the accuracy and *F1-score* is the harmonic mean of precision and sensitivity. These two are the major statistics to quantify the quality of the algorithm.

**Figure S1**. Illustration of calculating TP, FP, FN, and TN

Note that each block of “event” in **Figure S1** contains only one TP. This is because the classification precision (L = 50ms) is sufficiently short that only one heat beat can occur ( $\frac{L}{2}=25ms\ll RR interval)$.

1. *Optimal-threshold method*

The optimal threshold is calculated based on receiver operating characteristic (ROC) curve. Given each individual ECG signal recorded by textile sensors, the smoothed version of the energy is calculated by the third step of the proposed algorithm (see Methods). Given m = max (energy), 101 threshold values are selected within the interval of $[0,m]$ ($m_{i}=\frac{i}{100}*m, i=0,\ldots,100)$. TP, TN, FP and FN are calculated for each *m_i_*, and accordingly. TPR denotes “true positive rate” and FPR means “false positive rate”. ${TPR}_{i}=\frac{TP}{TP+FN} (sensitivity)$ and ${FPR}_{i}=\frac{FP}{FP+TN}$ ($1-specificity$) are computed to create the ROC curve. As shown in **Figure S2**, the optimal threshold is the *m_i_* with the maximum distance from the line$y \left( TPR \right)=x (FPR)$ in ROC curve.


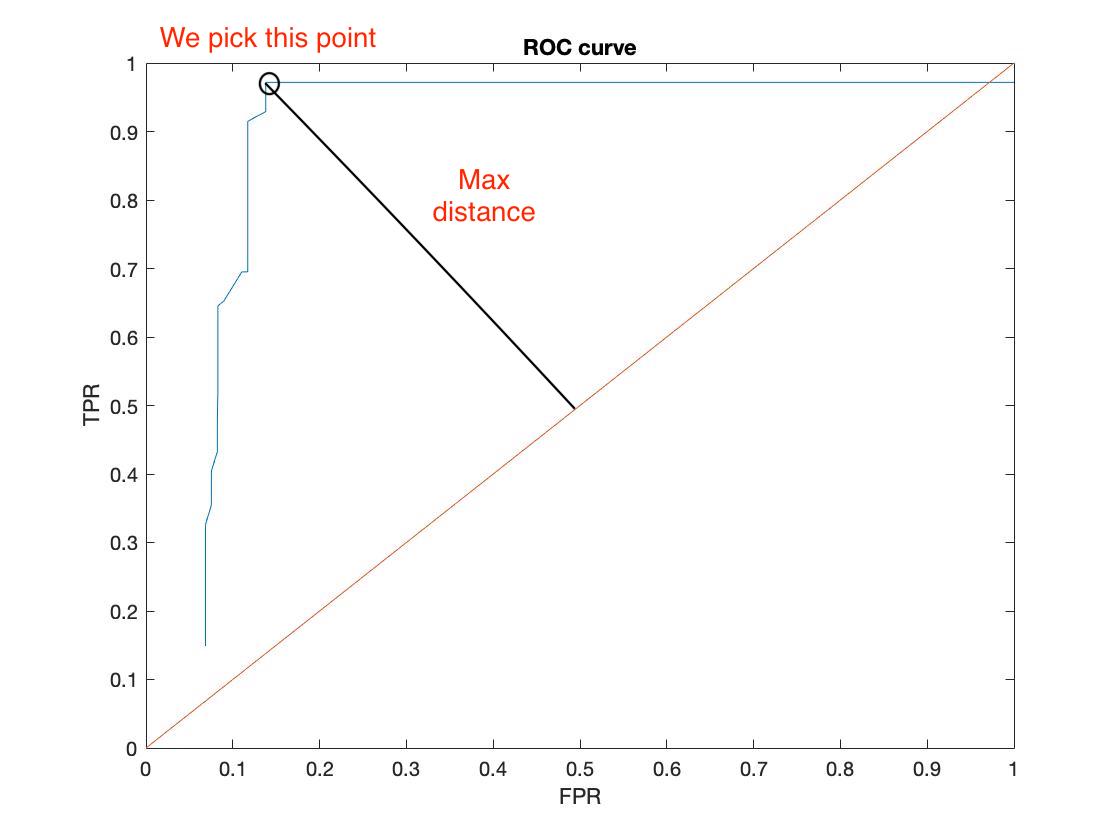


**Figure S2**: ROC curve and $m_{opt}$ choosing, high sensitivity case

It is to be noted that this optimum threshold is used in the 4^th^ step (peak detection) of the algorithm incorporating either HDIG or optimal-threshold methods.
